# Aorta Structural Alterations in Term Neonates: The Role of Birth and Maternal Characteristics

**DOI:** 10.1155/2013/459168

**Published:** 2013-07-25

**Authors:** Marco Matteo Ciccone, Pietro Scicchitano, Christian Salerno, Michele Gesualdo, Fara Fornarelli, Annapaola Zito, Lucia Filippucci, Roberta Riccardi, Francesca Cortese, Francesca Pini, Lucia Angrisani, Antonio Di Mauro, Federico Schettini, Nicola Laforgia

**Affiliations:** ^1^Cardiovascular Disease Section, Department of Emergency and Organ Transplantation, University of Bari, Piazza G. Cesare 11, 70124 Bari, Italy; ^2^Dipartimento di Medicina Traslazionale-Laboratorio Igiene ed Medicina Ambientale, University of Piemonte Orientale A. Avogadro, Via Duomo 6, 13100 Vercelli, Italy; ^3^Department of Cardiovascular Disease Rehabilitation, U.S.L.2, Via Guerra 21, 06127 Perugia, Italy; ^4^Neonatology and Neonatal Intensive Care Section, Department of Gynecology, Obstetrics and Neonatology, University of Bari, Piazza G. Cesare 11, 70124 Bari, Italy

## Abstract

*Aim*. To evaluate the influence of selected maternal and neonatal characteristics on aorta walls in term, appropriately grown-for-gestational age newborns. *Methods*. Age, parity, previous abortions, weight, height, body mass index before and after delivery, smoking, and history of hypertension, of diabetes, of cardiovascular diseases, and of dyslipidemia were all assessed in seventy mothers. They delivered 34 males and 36 females healthy term newborns who underwent ultrasound evaluation of the anteroposterior infrarenal abdominal aorta diameter (APAO), biochemical profile (glucose, insulin, total cholesterol, HDL and LDL cholesterol, triglycerides, fibrinogen, and D-dimers homeostasis model assessment [HOMA_IR_]index), and biometric parameters. *Results*. APAO was related to newborn length (*r* = +0.36; *P* = 0.001), head circumference (*r* = +0.37; *P* = 0.001), gestational age (*r* = +0.40, *P* = 0.0005), HOMA index (*r* = +0.24; *P* = 0.04), and D-dimers (*r* = +0.33, *P* = 0.004). Smoke influenced APAO values (odds ratio: 1.80; confidence interval 95%: 1.05–3.30), as well as diabetes during pregnancy (*r* = +0.42, *P* = 0.0002). Maternal height influenced neonatal APAO (*r* = +0.47, *P* = 0.00003). Multiple regression analysis outlined neonatal D-dimers as still significantly related to neonatal APAO values. *Conclusions*. Many maternal and neonatal characteristics could influence aorta structures. Neonatal D-dimers are independently related to APAO.

## 1. Introduction

Cardiovascular diseases (CVD) are still the major cause of death in developing countries, including China and India [1]. Primary prevention programs, instrumental evolution, pharmacological treatments, and lifestyle changes reduce CVD mortality compared to the 1960s data [2]. Nevertheless, CVD start since childhood. Since 1990s, the attention of physicians moved on the early stages of life [3]. The Cardiovascular Risk in Young Finns [3] was one of the first studies systematically evaluating the role of different conditions (i.e., family dietary habits and standard of education and diet of mothers) on the future cardiovascular risk profile of 3,596 children.

Dalziel et al. [4] demonstrated that adult patients born moderately preterm have increased blood pressure and insulin resistance at 30 years of age. Preterm birth rather than poor fetal growth seemed to be the major determinant of this association, although many epidemiological studies showed that low birth weight was independently associated with subsequent cardiovascular disease risk factors and increased incidence of cardiovascular disease in adulthood [5, 6]. The “fetal programming” during critical phases of development in utero could be responsible for metabolic disturbances in adult life and long-term structural changes of the vascular system [7–9].

Neonatal and maternal characteristics could early influence atherosclerosis development. Anteroposterior diameter of infrarenal abdominal aorta (APAO) is a reliable and well-established ultrasound parameter to detect early impairment of vascular structure since childhood [10, 11].

The aim of this study was to evaluate the influence of some selected maternal and neonatal characteristics on aorta walls (evaluated by mean of APAO) in term, appropriately grown-for-gestational age (AGA) newborns.

## 2. Methods

### 2.1. Subjects

Seventy mother-newborn pairs admitted, respectively, to the Obstetric Section and Neonatology and Neonatal Intensive Care of the Department of Gynecology, Obstetrics and Neonatology of the University of Bari were enrolled in the study.

The study was approved by the Institutional Review Board of Bari University General Hospital and carried out in accordance with the principles of the Helsinki Declaration.

#### 2.1.1. Newborns Characteristics

There were 34 males and 36 females healthy newborns, born at term (after 37 completed weeks of gestation), 38 by vaginal delivery, and 32 by C-section. Mean gestational age was 39.2 ± 1.2 weeks, mean birth weight 3, 289.3 ± 341.2 g (between 10th and 90th percentile as established by gender and gestational age-specific national charts [12], AGA), mean length 49.5 ± 2.2 cm, and head circumference 34.0 ± 1.2 cm. They all had normal transition (Apgar 5′ ≥  7). Newborns with any congenital malformations were excluded.

After informed consent, all newborns had ultrasound evaluation of the APAO, and biochemical profile was obtained on the blood taken at birth from umbilical cord. Blood samples had been performed early at morning (between 8 : 00 a.m. and 9 : 00 a.m.) after an overnight fast on the same day of the ultrasound.

APAO evaluations were done, within 24 hours after delivery, with the newborn in supine position, during a routine daytime rest period, in a warm environment (20-21°C). It was performed by a single operator using a single high-resolution vascular ultrasound Philips 5500 equipped with a 3 MHz electronic probe. To improve the image acquisition, neonates were examined at least 4–6 hours after breastfeeding to reduce intestinal bloating. The electronic probe was placed one centimeter left of the umbilicus. Then the best image in long-axis projection of the abdominal aorta was obtained. The APAO was defined as the maximal external cross-sectional diameter measurement of the infrarenal abdominal aorta. It was calculated as the distance between the near and the far walls of the abdominal aorta. Measurements were performed 1 cm above and distal to the umbilicus and expressed in centimeters [10, 11]. Repeatability and intraobserver variability of all ultrasound scan measurements were evaluated by means of intraclass correlation coefficient of >0.90. In particular, anteroposterior diameter showed good reproducibility with an intraclass correlation coefficient of 0.92.

Biochemical profile included glucose, insulin, total cholesterol, high-density lipoprotein (HDL) and low-density lipoprotein (LDL) cholesterol, triglycerides, fibrinogen, and D-dimers determinations. Insulin resistance was calculated by the homeostasis model assessment (HOMA_IR_) [13, 14].

#### 2.1.2. Maternal Characteristics

For each mother, data on age, parity, previous abortions, weight, height, and body mass index [weight (kg)]/[height (m)]^2^ before and after delivery were collected. Using a combination of clinical interviews and obstetric records, information about familiar history for hypertensive, diabetic, cardiovascular diseases, and dyslipidemic conditions were also obtained. Mothers were considered as smoker if they smoked at least five cigarettes/day within 3 months before delivery or if they stopped smoking <1 year before delivery. There were no inclusion criteria for the mothers, except to have delivered an AGA term newborn. 111 mothers and related newborns were consecutively enrolled, but 70 delivered AGA term newborn.

### 2.2. Statistical Analysis

The continuous variables were expressed as mean and standard deviation. 

Categorical data had been evaluated by means of odds estimation and CI 95 had been used in order to assess statistical significance. The Pearson's linear correlation coefficient was used to study the relationship between the continuous variables. Multiple regression analysis had been adopted in order to evaluate the influence of confounding factors on APAO evaluation. A *P* < 0.05 was considered statistically significant.

## 3. Results

Maternal mean age was 32 ± 5 years. Body mass index before delivery was within normal limits (22.75 ± 4.46 Kg/m^2^). There were 19 smokers (27%). Nine (13%) had gestational diabetes, and 6 (9%) and 3 (4%) haddyslipidemia. 22 (31%) had one or more previous abortions and 5 (7%) had 2 or more previous pregnancies. Familiarity for hypertension, cardiovascular diseases, dyslipidemic conditions, and diabetes are shown in Table 1.

Neonatal data are shown in Table 2. Mean APAO value was 4.76 ± 0.98 cm.

Pearson's linear regression has been used to assess any relationships between APAO values and maternal characteristics (Table 3). Furthermore, odds ratio and their relatives confidential intervals had been calculated for main dichotomous maternal variables considered (Table 4). Pearson's linear regression has been used to assess any relationships between APAO values and neonatal characteristics (Table 5).

APAO is directly related both to newborn length (*r* = +0.36; *P* = 0.001) and head circumference (*r* = +0.37; *P* = 0.001) at birth but not to birth weight (Table 5), and it is also strongly related to gestational age (*r* = +0.40, *P* = 0.0005).

**Figure 1:**
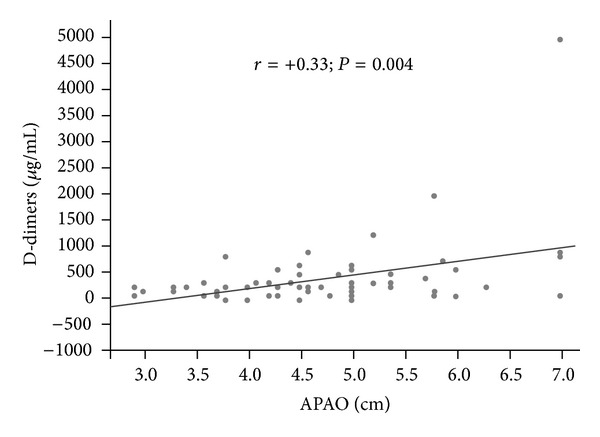
Linear regression analysis comparing anteroposterior abdominal aorta diameter and newborns D-dimers levels.

Interestingly, APAO is positively related to HOMA index (*r* = +0.24; *P* = 0.04), that is, an early marker of atherosclerosis (Table 5), while no correlation between APAO and insulin (*r* = +0.22; *P* = 0.056) was found. No correlations have been found between lipids blood levels and coagulation parameters (fibrinogen and prothrombin time-international normalized ratio [PT-INR]) with APAO values, but D-dimers (*r* = +0.33, *P* = 0.004) (Figure 1).

Important data come from the analysis of correlations between maternal characteristics and APAO. Smoking habit is a strong predictor of greater APAO values [Odds Ratio (OR): 1.80 (CI 95%: 1.05–3.30)]; thus, smoking could already exert a deleterious effect on the cardiovascular system of newborns. Even dyslipidemia familiarity seems to be slightly related to APAO increase [OR: 1.18 (CI 95%: 0.70–1.92)]. The same effect resulted for diabetes during pregnancy (*r* = +0.42, *P* = 0.0002).

Maternal height influences neonatal APAO (*r* = +0.47, *P* = 0.00003) while BMI before delivery and/or weight before delivery do not.

Because of multiple associations between maternal and neonatal characteristics with neonatal APAO, we performed a multivariate regression (Table 6). Only neonatal D-dimers were still significantly related to neonatal APAO values.

## 4. Discussion

In the last two decades, the scientific international community investigated the early phases of life as the moment from which atherosclerosis process could be outlined and, maybe, forced to reduce its development [3, 6]. The efforts to discover early markers of atherosclerosis since childhood lead to a new era of noninvasive technologies able to find the first moments of such a disabling process such as echocardiographic-Doppler evaluations [15], carotid intima-media thickness, flow-mediated vasodilatation, and APAO [10, 11, 16]. Our study aim was to evaluate the influence of maternal and neonatal characteristics on APAO in order to evaluate possible early factors of abdominal aorta walls alterations, that is, the beginning of atherosclerotic disease. APAO is an indicator of an earlier vascular remodeling and a high aortic stiffness, and it is therefore associated with a widespread vascular disease. It can be used to monitor patients at risk, even children [10, 11]. At the best of our knowledge, few studies have evaluated APAO in newborns.

Shankaran et al. [17] demonstrated a tight relationship between some maternal (smoke habits during pregnancy, alcohol intake, drug-addiction, etc.) and neonatal (birth weight, gender, etc.) characteristics with the cardiovascular risk profile evaluated at a 6-years followup. They were all term newborns, but the study showed the increase of blood pressure at 6 years of age only in those with intrauterine growth restriction (IUGR). Our study included only AGA infants and different maternal and neonatal characteristics, because it is already known that preterm delivery and/or IUGR are both factors able to increase the future cardiovascular risk [18, 19].

Some authors [20, 21] pointed out that maternal weight is a predictive factor for coronary heart diseases in adulthood and Poston [22] showed that gestational weight gain could influence long-term health of the neonate. Our study did not demonstrate a relationship between APAO and maternal weight and body mass index before delivery. We further showed a relationship between APAO and maternal height.

Our finding of a relationship between neonatal APAO and maternal smoking habits suggests that maternal smoking can cause an increased cardiovascular risk, already since birth [23–25]. Anna-Karin et al. [23] have found that maternal smoking is related to reduced descending aorta diameter in preterm newborns. Nevertheless, Anna-Karin et al. considered preterm newborns who had different physiological conditions than term neonates. Furthermore, their regression analysis did not consider all the confounding factors able to influence the predictability of the results, except gender and smoking. Differently from Anna-Karin et al., we have only evaluated term newborns, by considering all the parameter able to influence the relationship. At a linear analysis, smoking increased abdominal aorta intima-media thickness [24], probably through endocrine modifications, involving serum insulin-like growth factor-I (IGF-I) and insulin-like growth factor binding protein-3 (IGFBP-3) levels and actions [23] or by inducing a long-lasting “reprogramming” of the blood pressure control mechanisms [25], but the evaluation was not confirmed after adjustment for confounding variables. Nevertheless, as the hypothesis of Anna-Karin et al. about a reduced flow in placental circulation due to smoke could have influenced the reduced diameter found at abdominal aortic level, the same vascular alterations induced by smoke could enlarge the aortic diameter. For this reason further studies are needed in order to make such a matter clearer.

The relationship between APAO and coagulation parameters shows a statistically significant relationship between abdominal aorta diameter and D-dimers, the end product of stabilized fibrin degradation, whose values are higher in newborns compared to children and adults. D-dimers derive from the fibrin degradation through fibrinolysis process activation. It is encountered among those haemostatic tests able to improve the detection of coagulation process activation. Giannitsis et al. [26] already demonstrated the relationship between haemostatic factors and coronary artery diseases. Krupinski et al. [27] found an increased value of D-dimers in patients undergoing carotid endarterectomy: unstable plaques, in fact, seemed to show a higher fibrin intraplaque turnover able to induce an augmentation of D-dimers products, thus making D-dimers a surrogate marker of plaque instability. Clinical data recently indicated an association between elevated levels of D-dimers and the risk of subsequent clinical manifestations of cardiovascular disease [28]. The relationship between APAO and D-dimers in neonates is similar to the data from Empana et al. [28] in adults, and it has been confirmed by multivariate regression analysis. Thus, D-dimers could really become a marker of vessels walls instability and modification of their inner morphological structure since first days of life. At the best of our knowledge, no study have showed these results, but further studies are needed.

Furthermore, our data outlined a direct relationship between other neonatal parameters and APAO.

In particular, gestational age (*r* = +0.40; *P* = 0.0005), length (*r* = +0.36; *P* = 0.001), head circumference (*r* = +0.37; *P* = 0.001), and HOMA index (*r* = +0.24; *P* = 0.004) are all related to APAO.

Late gestational age is related to increased cardiovascular risk [29]. Our data seem to corroborate the literature findings: late gestational age is associated with increased cardiovascular risk. Nevertheless, biometrics in newborns' development is an important factor linked to cardiovascular risk. Touwslager et al. [30] demonstrated that birth weight, length, and head circumference of the infants were associated with impaired endothelial vasodilatation, that is, an early marker of atherosclerosis. Our findings seem to complete those of Touwslager et al.: we demonstrated that birth weight, length, and head circumference were all related to morphological alterations of the newborns' vasculature. This means that such neonatal parameters when altered could be considered as expression of morphological and functional early alterations of cardiovascular system; thus, they really increase cardiovascular risk profile of each individual.

In contrast with other studies [30, 31], we have not found that birth weight influences APAO.

APAO in our study population (term AGA newborns) is related to HOMA_IR_-index, similarly to what Simental-Mendía et al. [32] have demonstrated in small-for-gestational age (SGA) and large-for-gestational age (LGA). HOMA_IR_ index is a fundamental parameter calculated in order to evaluate one's insulin resistance condition. According to Gesteiro et al. data [33], our newborns showed normal values of HOMA_IR_ index in agreement with their age. Insulin resistance is already known to damage vessels walls by accelerating atherosclerotic process. Iannuzzi et al. [34] demonstrated an increased aortic stiffness related to HOMA_IR_ values. Nevertheless, these data involved obese children, while no data concerned newborns. At the best of our knowledge, this is the first study showing a direct relationship between insulin resistance increase and damages to aortic walls since the first moments of individuals' life. This means that physicians should better control pancreatic function of newborns in order to prevent further damages to cardiovascular system.

## 5. Conclusions

Despite the small sample size, we obtained a preliminary evaluation of the relationship between maternal and neonatal characteristics and the cardiovascular system of the newborn, evaluated as APAO, an excellent, noninvasive and reproducible tool able to evaluate the cardiovascular risk profile in newborns. Further researches are needed in order to improve our findings.

## Figures and Tables

**Table 1:** Maternal characteristics.

	Values (%)
Total number	70
Age (years)	32 ± 5
Familiarity for diabetes	
None	32 (46)
I degree relatives	10 (14)
II degree relatives	28 (40)
Familiarity for cardiovascular diseases	
None	39 (56)
I degree relatives	19 (27)
II degree relatives	12 (17)
Familiarity for hypertension	
None	35 (50)
I degree relatives	27 (39)
II degree relatives	8 (11)
Familiarity for dyslipidemia	
None	47 (67)
I degree relatives	17 (24)
II degree relatives	6 (9)
Previous abortions	22 (31)
No. of pregnancy >2	5 (7)
Smoking	19 (27)
Hypertension	6 (9)
Diabetes	9 (13)
Dyslipidemia	3 (4)
BMI before pregnancy (Kg/m^2^)	22.75 ± 4.46
Weight before pregnancy (Kg)	60.93 ± 11.72
Weight after pregnancy (Kg)	73.00 ± 11.79
Height before pregnancy (cm)	163.81 ± 6.58

Values are expressed as numbers and related percentages. BMI: body mass index.

**Table 2:** Neonatal characteristics.

	Values (%)
Total number	70
Male gender	34 (49)
Gestational age (weeks)	39.2 ± 1.2
Weight at delivery (gr)	3289.3 ± 341.2
Length at delivery (cm)	49.5 ± 2.2
Head circumference (cm)	34.0 ± 1.2
APAO (cm)	4.76 ± 0.98
D-dimers (*µ*g/mL)	332.5 ± 648.5
PT-INR	1.6 ± 0.7
Fibrinogen (mg/dL)	210.9 ± 49.5
APGAR 5′	9.7 ± 1.1
Glycemia (mg/dL)	48.9 ± 25.2
Insulin (*µ*UI/mL)	3.8 ± 1.4
HOMA index	0.59 ± 0.98
Total cholesterol (mg/dL)	73.9 ± 27.2
LDL-cholesterol (mg/dL)	29.3 ± 14.0
HDL-cholesterol (mg/dL)	30.6 ± 12.6
Triglycerides (mg/dL)	70.9 ± 45.5
Spontaneous delivery	38 (54)
Caesarean delivery	32 (46)

Values are expressed as numbers and percent. APAO: anteroposterior abdominal aorta diameter; APGAR 5′: American Pediatric Gross Assessment Record at 5 minutes; HDL: high-density lipoprotein; HOMA: homeostatic model assessment; LDL: low-density lipoproteins; PT-INR: prothrombin time-international normalized ratio.

**Table 3:** Linear correlation analysis between neonatal antero-posterior abdominal aorta diameter (APAO) and main maternal variables considered.

Maternal variables	Correlation coefficient (*r*)	*P* value
Mean age	+0.22	0.06
Total amount of pregnancy		0.19
Eclampsia		0.98
Hypertension		0.97
Diabetes	**+0.42**	**0.0002**
Dyslipidaemia		0.98
BMI before pregnancy		0.45
BMI after delivery		0.21
Height	**+0.47**	**0.00003**
Weight before delivery		0.16
Weight after delivery	**+0.24**	**0.04**

APAO: anteroposterior abdominal aorta diameter; BMI: body mass index; CVD: cardiovascular diseases.

**Table 4:** Odds ratio evaluations between neonatal antero-posterior abdominal aorta diameter (APAO) and main dichotomous maternal variables considered.

Findings	Odds ratio	95% CI
Smoke	1.80	1.05–3.30
CVD familiarity	0.78	0.47–1.29
Diabetes familiarity	0.59	0.35–1.01
Dyslipidaemia familiarity	1.18	0.70–1.92
Hypertension familiarity	0.82	0.50–1.35

CI: confidential interval; CVD: cardiovascular disease.

**Table 5:** Linear correlation analysis between neonatal antero-posterior abdominal aorta diameter (APAO) and main neonatal variables considered.

Neonatal variables	Correlation coefficient (*r*)	*P* value
Gestational age	**+0.40**	**0.0005**
Weight		0.77
Length	**+0.36**	**0.001**
Head circumference	**+0.37**	**0.001**
PT-INR		0.17
Fibrinogen		0.24
D-dimers	**+0.33**	**0.004**
APGAR 5′		0.12
Glycemia		0.18
Insulin	+0.22	0.056
HOMA index	**+0.24**	**0.04**
HDL-cholesterol		0.35
LDL-cholesterol		0.56
Total-cholesterol		0.52
Triglycerides		0.56

APAO: anteroposterior abdominal aorta diameter; APGAR 5′: American Pediatric Gross Assessment Record at 5 minutes; HDL: high-density lipoprotein; HOMA: homeostatic model assessment; LDL: low-density lipoproteins; PT-INR: prothrombin time-international normalized ratio.

**Table 6:** Multivariate correlation analysis between neonatal antero-posterior abdominal aorta diameter (APAO) and main maternal and neonatal variables considered.

	Coefficient	Standard error	*P* value
Model 1			
Maternal variables			
Maternal age	−0.006	0.023	0.808187
BMI-after	0.018	0.029	0.538889
Height	0.031	0.020	0.119716
Smoke	0.337	0.283	0.239169
CVD familiarity	0.022	0.180	0.901925
Diabetes familiarity	−0.169	0.150	0.264693
Dyslipidaemia familiarity	0.051	0.212	0.810230
Hypertension familiarity	−0.051	0.205	0.803890
Eclampsia	−0.019	0.531	0.970903
Total pregnancies	−0.188	0.157	0.237714
Type of delivery (spontaneous/caesarean)	−0.218	0.220	0.324930

Model 2			
Neonatal variables			
Gestational age	0.053	0.117	0.651170
Length	−0.029	0.059	0.632319
Weight	0.000	0.000	0.553384
Head circumference	−0.051	0.111	0.649230
PT-INR	0.105	0.174	0.548517
D-dimers	**0.001**	**0.000**	**0.010114**
Fibrinogen	−0.001	0.003	0.827094
Total cholesterol	−0.049	0.055	0.375519
HDL-cholesterol	0.029	0.055	0.594972
LDL-cholesterol	0.055	0.058	0.347597
Triglycerides	0.009	0.012	0.441564
Glycemia	−0.004	0.007	0.518465
Insulin	−0.048	0.090	0.593545
HOMA index	0.153	0.498	0.759908

APAO: anteroposterior abdominal aorta diameter; BMI: body mass index; CVD: cardiovascular diseases; HDL: high-density lipoprotein; HOMA: homeostatic model assessment; LDL: low-density lipoproteins; PT-INR: prothrombin time-international normalized ratio.
